# The Transcription Factor TCF1 in T Cell Differentiation and Aging

**DOI:** 10.3390/ijms21186497

**Published:** 2020-09-05

**Authors:** Chulwoo Kim, Jun Jin, Cornelia M. Weyand, Jörg J. Goronzy

**Affiliations:** 1 Division of Immunology and Rheumatology, Department of Medicine, Stanford University, Stanford, CA 94305-5166, USA; cwkim@stanford.edu (C.K.); jinjun@stanford.edu (J.J.); cweyand@stanford.edu (C.M.W.); 2 Department of Medicine, Palo Alto Veterans Administration Healthcare System, Palo Alto, CA 94304, USA

**Keywords:** TCF1, WNT/β-catenin, T cell aging, T cell differentiation, memory T cells, T follicular helper cells, T cell exhaustion, stem-like CD8 T cells, immunosenescence

## Abstract

The transcription factor T cell factor 1 (TCF1), a pioneer transcription factor as well as a downstream effector of WNT/β-catenin signaling, is indispensable for T cell development in the thymus. Recent studies have highlighted the additional critical role of TCF1 in peripheral T cell responses to acute and chronic infections as well as cancer. Here, we review the regulatory functions of TCF1 in the differentiation of T follicular helper cells, memory T cells and recently described stem-like exhausted T cells, where TCF1 promotes less differentiated stem-like cell states by controlling common gene-regulatory networks. These studies also provide insights into the mechanisms of defective T cell responses in older individuals. We discuss alterations in TCF1 expression and related regulatory networks with age and their consequences for T cell responses to infections and vaccination. The increasing understanding of the pathways regulating TCF1 expression and function in aged T cells holds the promise of enabling the design of therapeutic interventions aiming at improving T cell responses in older individuals.

## 1. Introduction

T cell factor 1 (TCF1, encoded by *TCF7*) was discovered in studies aiming at identifying regulators of human T lymphocyte development [1]. While involved in promoting embryonic stem cell self-renewal in almost all organs during murine embryogenesis, it is exclusively expressed in T lymphoid tissues postnatally [2,3]. In early thymocyte development, TCF1 is induced by NOTCH signaling and functions as a pioneer transcription factor initiating the T cell lineage program through genome-wide changes in chromatin accessibility [4]. TCF1 deficiency causes multiple defects on the path from the double-negative to the double-positive stage of T cell development, impairing thymocyte maturation [5]. The double-positive/single-positive transition is also impaired in TCF1-deficient mice, indicating that TCF1 is important to mediate apoptosis and negative selection of thymocytes [6]. Enforced TCF1 expression in bone marrow progenitors was sufficient to induce many T cell identity genes, in particular the expression of transcription factors essential for T cell commitment and specification such as *Gata3* and *Bcl11b* in mice [7]. 

TCF1 was discovered in 1996 to function as a direct effector downstream of the WNT/β-catenin signaling pathway [8] that plays essential roles in embryonic development [9] and stem cell self-renewal in adult tissues [10]. WNT signaling is initiated by the binding of an extracellular WNT-protein ligand to a cell surface Frizzled family receptor. This binding induces an intracellular signaling cascade that prevents GSK-3β-mediated β-catenin phosphorylation and proteasomal degradation, allowing β-catenin to accumulate in the cytoplasm and translocate into the nucleus. The nuclear β-catenin binds to and acts as a co-activator of TCF1, enabling the recognition of TCF1-specific sequence motifs in promoters and enhancers of WNT target genes [11]. TCF1 exists as long and short isoforms. The long TCF1 contains a β-catenin-binding domain and promotes transcription of WNT target genes upon binding with β-catenin; the short, “dominant-negative” TCF1 lacks the binding domain and inhibits the ability of overexpressed WNT or β-catenin to induce gene expression [11]. The β-catenin/TCF1 interaction enhances TCF1-dependent transcriptional activity in thymocytes and is required for full thymocyte development [12].

Beyond thymic development, WNT signaling as well as TCF1-regulated expression of transcription factors and T cell identity genes play a role in the differentiation of mature T cells. Here, we will review recent insights into the mechanisms of how TCF1 regulates T cell fate in response to acute and chronic infections and discuss the implications of TCF1 function for T cell exhaustion and T cell aging.

## 2. TCF1 in T Follicular Helper Cell Differentiation

In immune responses to infections, naïve CD4 T cells undergo robust expansion and differentiate into functionally distinct T helper cells, such as TH1 and TH17 effector T cells that coordinate pathogen clearance, or T follicular helper (TFH) cells that help B cell responses. TFH cells express the chemokine receptor CXCR5 that, in response to chemokine CXCL13, facilitates migration to B cell follicles. They provide help to cognate B cells to differentiate into high-affinity antibody-producing plasma cells and memory B cells via production of cytokines such as IL-21 and IL-4 and stimulation with CD40L and inducible T cell co-stimulator (ICOS) [13]. For most effector CD4 T cell populations, differentiation is guided by specific cytokines that activate specific transcription factor networks. TFH cell generation is more permissive with different combinations of signals being effective [14]. 

**Figure 1:**
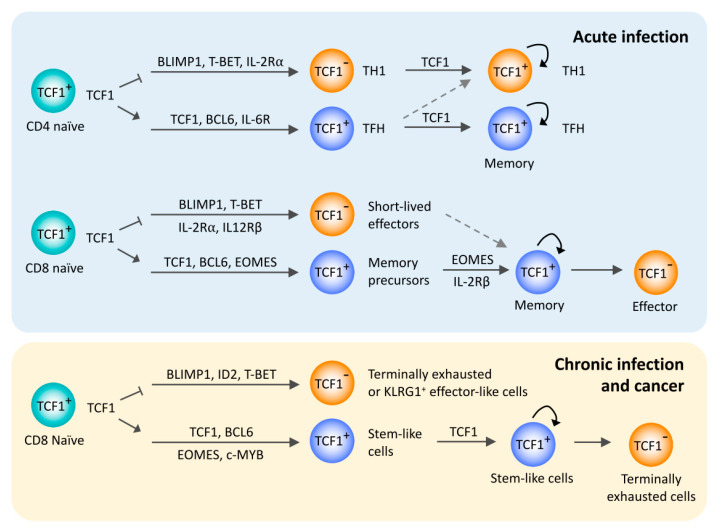
TCF1 in peripheral T cell responses to acute and chronic antigen stimulation. Overview of the regulatory functions of TCF1 in the differentiation of T follicular helper cells and memory T cells after acute infections and stem-like exhausted CD8 T cells after chronic infection or cancer. TCF1 is involved in a common gene regulatory network across these T cell subsets, mainly by promoting BCL6 expression and repressing BLIMP1 expression.

While B cell lymphoma 6 (BCL6) is the transcription factor absolutely required for the generation and function of TFH cells, TCF1 is essential for the early steps in TFH differentiation (Figure 1) [13]. BCL6 functions in part through repressing the expression of T-BET (encoded by *TBX21*) and B lymphocyte-induced maturation protein 1 (BLIMP1, encoded by *PRDM1*), the transcription factors guiding differentiation to TH1 cells [15,16,17]. BCL6 also represses the expression of other transcriptional repressors for TFH cells such as RUNX3 and GATA3 [18]. Conversely, T-BET together with BLIMP1 is a strong negative regulator of TFH cell differentiation by repressing BCL6 and TFH-associated gene expression such as CXCR5. T-BET also directly binds to BCL6 and abrogates its DNA binding activity, which inhibits BCL6-dependent gene repression [19,20].

TCF1 is highly expressed in TFH cells after viral and bacterial infections in mice. Deletion of TCF1 impaired TFH cell generation and development of germinal center B cells [21,22,23]. Of note, TFH differentiation was particularly impaired when TCF1 was deficient in conjunction with the closely related lymphoid enhancer binding factor-1 (LEF1), both of which are members of the high-mobility group (HMG) of transcription factors [21]. Conversely, ectopic expression of TCF1 or LEF1 increased TFH cell differentiation and induced TFH-related genes in TH1 cells such as CXCR5 and programmed cell death protein 1 (PD-1) [21,22,23]. Mechanistically, TCF1 increases BCL6 expression by binding to its transcription start site while repressing BLIMP1 and IL-2Rα expression. IL-6 is an early regulator of TFH differentiation, and TCF1 directly induces the expression of IL-6 receptors. BLIMP1 induced by IL-2/signal transducer and activator of transcription 5 (STAT5) signaling reciprocally represses transcription of TCF1 and BCL6. Overexpression of BCL6 rescues defective TFH cell differentiation of TCF1-deficient CD4 T cells, indicating that TCF1 acts upstream of BCL6-BLIMP1 cross-regulation [21,22,23].

Studies have shown that TFH effector cells persist after peak responses and develop into TFH memory cells (Figure 1) [24,25,26]. CXCR5^+^ memory CD4 T cells preserve transcriptomic and phenotypic TFH signatures. After adoptive transfer into new hosts and re-exposure to an antigen, CXCR5^+^ memory CD4 T cells primarily had a TFH effector cell phenotype and rapidly provided help to B cells [25]. The factors controlling this memory transition are not understood but appear to again include TCF1 function [27]. This role of TCF1 may not be limited to TFH cells but also pertinent for memory T cell generation in general. TCF1 deficiency impaired the generation of all memory CD4 T cells following viral infection, although CXCR5^+^ TFH memory cells are much more affected than TH1 memory cells [28]. As will be alluded to in more detail in the next chapter, signaling and transcription factor networks favoring TFH cells or memory cells of other T cell lineages exhibit similarities [24]. IL-2 signaling through STAT5 is a strong negative regulator of TFH cell differentiation [29,30]. TFH memory CD4 T cells are largely derived from CD25^low^ effector CD4 T cells generated during early cell divisions after viral infection, as are TH1 memory cells [31]. Given that IL-2-induced BLIMP1 represses transcription of BCL6 and TCF1 [23,29], increased TCF1 expression in CD25^low^ early effector CD4 T cells is essential for the generation of memory CD4 T cells. Moreover, TCF1 appears to be pivotal in the asymmetric division model of memory cell generation (i.e., a cell division produces two daughter cells committed to distinct differentiation states). Offspring of influenza-specific CD4 T cells lost TCF1 expression after few divisions and then developed into tissue-infiltrating effector T cells, while those that maintained TCF1 expression homed to non-draining lymph nodes and developed memory and TFH-like features [32]. Based on in vitro models, this asymmetric development of daughter cells was attributed to the unequal allocation of phosphoinositide 3-kinase (PI3K)/mammalian target of rapamycin (mTORC) signaling molecules that induced distinct metabolic states in the two offspring [32].

## 3. TCF1 in CD8 T Memory Cell Differentiation

Similar to CD4 TFH cells, TCF1 is a key component of the regulatory networks that favor the generation of memory CD8 T cells (Figure 1). CD8 T cells are critical for eliminating intracellular pathogens. More clearly than for CD4 T cells, the CD8 T cell response has been phenotypically dissected into short-lived effector (SLEC) and memory precursor (MPEC) cells [33]. At the peak of a T cell response to acute infections, antigen-specific CD8 T cells represent a heterogeneous population of effector and memory precursor cells. Most effector cells express killer cell lectin-like receptor G1 (KLRG1), are short-lived and produce high amounts of inflammatory cytokines and cytolytic molecules, whereas MPEC highly expresses IL-7Rα [34,35]. A recent study showed additional heterogeneity within the KLRG1^+^ effector CD8 T cell population, which contains a subset that also contributes to the memory pool [36]. Transcription factor networks accounting for the fate decision have been identified [33,37]. T-BET [38], BLIMP1 [39,40], Inhibitor of DNA binding 2 (ID2) [41] and Zinc-finger E-box binding homeobox 2 (ZEB2) [42] promote the differentiation to short-lived effector cells, whereas generation of memory precursors and central memory CD8 T cells is directed by BCL6 [43], Eomesodermin (EOMES) [44], Forkhead box O1 (FOXO1) [45,46,47], BTB domain and CNC homolog 2 (BACH2) [48] and ID3 [41].

TCF1 is highly expressed in naïve CD8 T cells and downregulated during effector differentiation in part due to the inflammatory cytokine IL-12-induced STAT4 activation [49]. Developing memory cells re-express TCF1, suggesting a role of TCF1 in memory CD8 T cell differentiation [50,51]. In response to acute viral and bacterial infection in mice, TCF1-deficient CD8 T cells had a defect in the generation of MPEC and CCR7^+^ CD62L^+^ central memory T cells [52,53]. Once generated, TCF1-deficient memory precursor cells abundantly expressed transcription factors for effector cell programming including BLIMP1, T-BET, ID2 and ZEB2 [49]. Additionally, secondary expansion of TCF1-deficient CD8 memory T cells was severely impaired, leading to reduced protection upon secondary challenge [52,53]. In humans, memory CD8 T cells in blood, spleen and lymph nodes express a variable amount of TCF1, with expression levels positively correlating to their proliferative potential [54,55]. TCF1 expression is essential for the longevity of memory CD8 T cells. TCF1 binds to the regulatory region of *Eomes* and increases its expression, which in turn induces IL-2Rβ expression important for IL-15-dependent homeostasis [52]. Given that TFH cells and memory precursor CD8 T cells have a similar gene regulatory network for their differentiation [24], the mechanisms regarding how TCF1 affects the generation of memory CD8 T cells are likely similar to that in TFH cells, including repression of T-BET and BLIMP1 transcription and induction of BCL6 [49,56]. Interestingly, a recent study found that TCF1 and LEF1 have histone deacetylase activity and therefore can change the posttranslational modification of histones to a more repressive state. This mechanism may contribute to a repression of CD4 T cell-related genes in naïve CD8 T cells such as *Cd4* and *Foxp3*. Moreover, TCF1 also binds to several effector genes including *Prdm1* and removes acetylation [57]. Whether the histone deacetylase activity of TCF1 is involved in memory T cell generation remains to be examined.

The long isoform of TCF1 is an effector target of the WNT/β-catenin pathway. β-catenin bound to the long TCF1 isoforms acts as a co-activator of TCF1-mediated transcription [11]. This raises the possibility that WNT signaling is involved in regulating the generation of memory CD8 T cells. Overexpression of the TCF1 long isoforms containing the β-catenin binding domain, but not the short isoform rescued a defect in the secondary expansion of TCF1-deficient memory CD8 T cells [53]. Further, enforced expression of both the TCF1 long isoform and stabilized β-catenin increased the generation of memory CD8 T cells [51]. These data suggest that the WNT/β-catenin pathway is involved in memory generation through the long isoform of TCF1. In support, pharmacological inhibition of glycogen synthase kinase 3β (GSK-3β) or stimulation with the recombinant WNT3a during T cell priming activated the WNT/β-catenin pathway and promoted memory T cell generation with increased multipotent and self-renewing stem cell-like features [58]. However, data conflicting with this model have also been described, and a unifying model is currently elusive. One study showed that conditional knockout of β-catenin in peripheral CD8 T cells did not alter the generation of memory CD8 T cells after viral infection or recall response [59]. In another study, selective deletion of the TCF1 long isoforms without altering the short isoforms did not reduce the generation of memory CD8 T cells, although development of central memory cells and recall expansion were modestly reduced [28].

## 4. TCF1 in T Cell Exhaustion in Chronic Viral Infection and Cancer

Prolonged exposure to an antigen during chronic viral infections or cancer induces exhaustion in the responding CD8 effector T cell populations, setting them apart from effector or memory CD8 T cells generated during acute infections [60]. Exhausted CD8 T cells are characterized by progressive loss of effector functions, upregulation of multiple inhibitory receptors, such as PD-1, cytotoxic T lymphocyte antigen 4 (CTLA-4) and T cell immunoglobulin- and mucin-domain–containing molecule-3 (TIM3), and poor proliferative capacity [61]. While development of T cell exhaustion protects hosts from excessive tissue damage under persistent antigen stimulation, it also limits anti-viral or anti-tumor T cell responses and leads to the persistence of disease. Blocking checkpoint-inhibitory receptors on exhausted CD8 T cells, including PD-1 and CTLA-4, enhances the proliferation and function of some exhausted T cells, leading to a reduction in viral load or suppression of cancer load [60,62].

Exhausted CD8 T cells comprise heterogeneous cell populations, with TCF1 being an important determinant in this heterogeneity (Figure 1). Studies of chronic lymphocytic choriomeningitis virus (LCMV) infection in mice have identified a subset of PD-1^+^ CD8 T cells that have self-renewal capacity, therefore also called stem-like CD8 T cells [63,64,65,66,67]. This subset expresses the transcription factors TCF1, LEF1, BCL6 and ID3, the costimulatory molecules CD28 and ICOS and the chemokine receptors CXCR5 and CCR7, but lacks the inhibitory receptor TIM3. Upon adoptive transfer, TCF1^+^ CD8 T cells lose TCF1 expression and upregulate BLIMP1, PD-1 and TIM3, giving rise to terminally differentiated exhausted CD8 T cells. Importantly, TCF1^+^ CD8 T cells represent the population that is responsive to PD-1 checkpoint blockade and that accounts for the improved control of viral load [63,64,66].

TCF1^+^ PD-1^+^ CD8 T cells have a transcriptional signature characteristic of TFH cells or memory precursor CD8 T cells generated following acute infections. In contrast, terminally differentiated exhausted cells have phenotypic and transcriptomic signatures characteristics of TH1 cells or KLRG1^+^ terminal effector CD8 T cells generated after acute infections [63]. However, both effector T cell types generated during chronic infection are epigenetically clearly distinct from those developed after acute infection [68,69,70], suggesting that exhaustion represents a unique state of T cell differentiation rewired in responses to chronic antigen stimulation. Similar to the transcriptome, the chromatin landscape of TCF1^+^ stem-like CD8 T cells is fundamentally different from that of terminally exhausted CD8 T cells, supporting the notion that stem-like CD8 T cells are a distinct population [70,71,72,73]. Recent single-cell transcriptome analysis of TCF1^+^ CD8 T cells from mice after acute and chronic viral infections revealed that their transcriptional programs diverged as early as on day 7 after infection [74].

TCF1^+^ antigen-specific CD8 T cells have been found in human settings of chronic infection such as chronic hepatitis C [66,75], hepatitis D virus [76] and human immunodeficiency virus [64,65] as well as in malignant diseases, such as melanoma [67,71,72,77], non-small cell lung cancer [78] and renal cancer [73]. Importantly, their cell frequencies positively correlated with clinical outcomes and overall survival. Functionally, while TCF1^–^ terminally exhausted tumor-infiltrating CD8 lymphocytes (TILs) expressed more interferon-γ (IFN-γ) and granzyme B, TCF1^+^ stem-like TILs were more polyfunctional, as shown by co-producing IFN-γ, tumor necrosis factor α (TNFα) and IL-2 [71,78]. TCF1^+^, but not TCF1^–^ TILs proliferated more and gave rise to both TCF1^+^ and TCF1^–^ CD8 T cells in response to PD-1 blockade, leading to significantly better and long-term control of tumor growth as compared to TCF1^–^ TILs [71,77].

Transcription factor networks involved in the diversification of exhausted T cells, at least in part, resemble those distinguishing effector from memory or TFH cells in acute infection [63], while the transcription factor thymocyte selection-associated high mobility group box (TOX) appears to be an essential regulator for inducing exhausted T cells in general [74,79,80]. TCF1 is critical for the development of stem-like progenitor cells [63,65,66,67,81], while T-BET, BLIMP1 and EOMES contribute to controlling the degree of exhaustion during chronic infection [61,82,83,84]. Moreover, T cell receptor (TCR) stimulation induces a transcription factor network of IRF4 together with BATF and NFAT that represses TCF1 expression and impairs the generation of stem-like CD8 T cells [85]. TCF1-deficient CD8 T cells expanded after chronic infection but failed to generate stem-like progenitor cells, leading to a rapid loss of CD8 T cells and an impairment of viral control [63,65]. Similarly, in a mouse model of melanoma and vaccination with tumor antigen, TCF1-deficient CD8 T cells were initially abundant in tumors and tumor-draining lymph nodes but rapidly declined due to the lack of stem-like TILs, leading to reduced long-term tumor control [77]. Conversely, ectopic expression of TCF1 enhanced the development of stem-like CD8 T cells that persisted long-term in mice with chronic LCMV infection [67]. Consistent with the function of TCF1 in promoting the differentiation of TFH and memory precursor CD8 T cells following acute infection, TCF1 controls BCL6 and BLIMP1 expression in exhausted CD8 T cells by directly binding to the promoter region of *Bcl6* and the regulatory site of *Prdm1* [67]. Ectopic expression of BCL6 showed similar results to TCF1 overexpression, supporting the notion that TCF1 acts upstream of BCL6 [67]. Additionally, early after chronic infection, TCF1 represses an opposing cell fate differentiation into KLRG1^+^ effector-like cells by antagonizing *Id2*, *Prdm1* and *Runx1* expression [81]. TCF1 also promotes EOMES expression, which in turn induces the c-MYB transcription factor required for BCL2 expression and cell survival [81]. In addition to TCF1, stem-like T cells are different from exhausted cells in that they have increased chromatin accessibility to NF-κB-p65 motifs while closing sites harboring ETS motifs. This feature also sets them apart from unstimulated naïve or memory cells after acute viral infection that both share the high accessibility to TCF motifs [70]. Taken together, TCF1 is essential for the differentiation of stem-like CD8 T cells under conditions of chronic infection, which provide long-term persistence of a pool of exhausted CD8 T cells during chronic viral infection or cancer (Figure 1). Moreover, TCF1 appears to be a critical regulator during the progressive differentiation to exhausted CD8 T cells [86].

## 5. TCF1 in T Cell Aging

**Figure 2:**
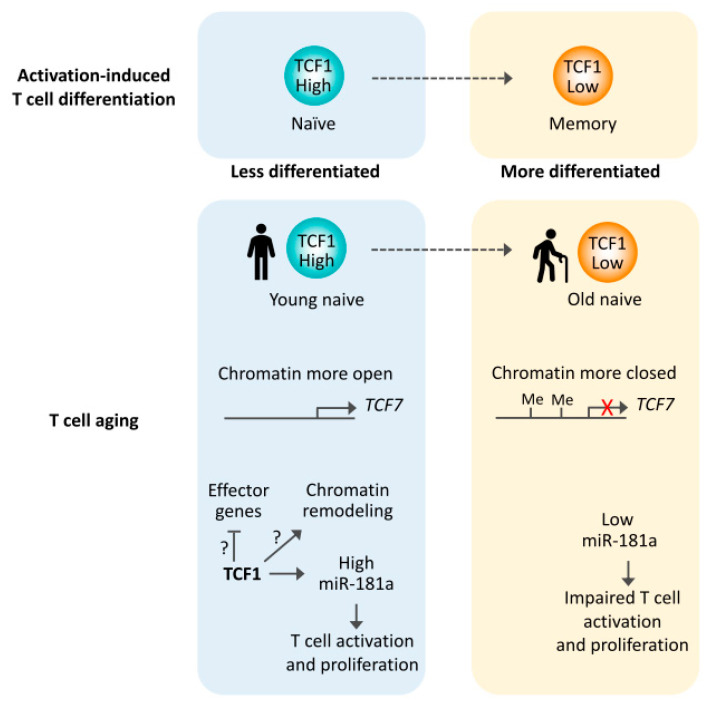
Reduced TCF1 expression in T cell aging. Activation-induced T cell differentiation from naïve to memory T cells is coupled with reduced TCF1 expression. Similar to memory T cells, aged naïve T cells have reduced TCF1 expression, at least in part due to reduced chromatin accessibility and increased DNA methylation at the *TCF7* gene. miR-181a is one TCF1 targets in naïve T cells, with miR-181a levels being critical for protective antiviral immune response.

Given that TCF1 is maintaining stem-like properties in memory T cells after acute infection as well as in PD-1^+^ CD8 T cells in chronic infection and cancer, it is not surprising that TCF1 is also involved in the aging process. Aging of the immune system induces defects in particular of the adaptive immune response that are only to a lesser extent explained by the failed replenishment of T cells due to thymic involution and that result in impaired control of acute and latent viral infections and diminished vaccine responses [87]. Possible mechanisms have been the subject of recent reviews [88,89,90]. In peripheral blood mononuclear cells (PBMC) from older adults, chromatin accessibilities at regulatory regions of the *TCF7* as well as the *LEF1* gene are reduced, which correlates to their decreased gene expression [91]. Moreover, the *TCF7* gene is hypermethylated and less expressed in CD8 T cells from older individuals [92]. Since these studies did not distinguish between naïve and memory cells, the epigenetic and transcriptional changes could reflect population shifts towards effector T cells, as it occurs in particular in CD8 T cells [93,94]. Indeed, the *Tcf7* locus has more repressive histone marks (H3K27me3) and DNA methylation but less active histone marks (H3K27ac) in SLECs compared to MPECs in mice [95,96,97]. Further, as human naïve as well as memory CD8 T cells are becoming more differentiated with older age, the *TCF7* gene promoter progressively acquires more DNA methylation, accounting for reduced gene expression [98]. In agreement, our preliminary studies show that naïve CD4 T cells from older individuals have reduced TCF1 expression compared to young adults (Ye et al., submitted) (Figure 2). TCF1, in conjunction with the transcription factor Yin Yang-1 (YY1), is a transcriptional activator of *pri-miR-181a*, whose expression in naïve CD4 and CD8 T cells declines with age [99,100]. Activation of WNT/β-catenin signaling via GSK-3β inhibitors in vitro increases miR-181a expression and enhances TCR signaling in naïve T cells from old individuals (Ye et al., submitted). miR-181a controls TCR activation thresholds by targeting several phosphatases downstream of TCR, including PTPN22, SHP2, DUSP5 and DUSP6 and therefore reducing the expression of these negative regulators of TCR signaling [101]. Increased expression of miR-181a reduces DUSP6 protein expression, which in turn restores extracellular signal-regulated kinase (ERK) signaling and TCR activation. The functional importance of this age-associated miR-181a loss has been proven in a mouse model of an acute viral infection. Conditional deletion of miR-181ab1 in peripheral T cells impaired the expansion of virus-specific CD8 T cells after acute LCMV infection and delayed viral clearance [102], mirroring T cell defects seen in old individuals in responses to infections such as with yellow fever, West Nile virus and likely the current SARS-CoV-2 [103,104,105,106]. miR-181a deficiency also reduced the generation of memory cells particularly in the liver and abrogated secondary expansion of memory cells [102].

**Figure 3:**
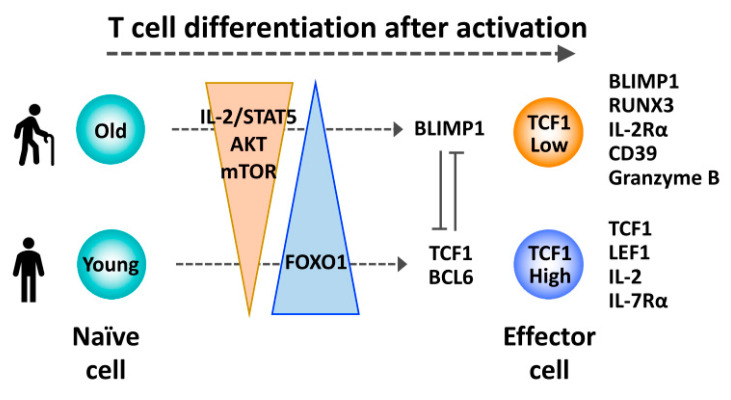
Reduced TCF1 expression promoting a differentiation bias of activated aged T cells toward terminal effector cells. After priming, increased IL-2/STAT5 signaling and sustained activation of the AKT-mTOR pathway favors differentiation of aged T cells into TCF1^low^ terminal effector cells, due to reduced FOXO1/TCF1 expression and increased BLIMP1 expression.

The loss of TCF1 expression may contribute to the loss of stemness in naïve T cells from older individuals (Figure 2). Signatures of differentiation have been one of the epigenetic hallmarks of T cell aging [107,108]. The TCF1 target, miR-181a, declines in more differentiated memory T cells as well as with age [99]. Moreover, the histone deacetylase activity of TCF1 represses effector gene expression, including *Prdm1*, in naïve CD8 T cells [57]. Preferential differentiation into terminally differentiated effector T cells is one of the key features of old naïve T cells [109,110]. Upon activation, proliferating old T cells have reduced expression of TCF1 and LEF1 compared to young T cells (Figure 3). They instead highly express transcription factors BLIMP1, JUN and RUNX3 [109]. Consistent with this transcription factor network, activated old T cells have a more pronounced effector cell profile, including higher expression of IL-2Rα, TIM3 and granzyme B but reduced expression of CD28, IL-7Rα and IL-2 [109]. BCL6 is an important repressor of the ectoATPase CD39 that is accordingly increased in T cell responses of older adults and, in a positive feedback loop, further deviates T cell differentiation from TFH cells or MPECs to SLECs [110,111]. Moreover, the ATPase activity and the corresponding generation of adenosine contributes to the rapid loss of SLECs after peak responses [110]. Further, aged T cells have a higher expression of transforming growth factor β (TGFβ) receptor than young T cells. When activated with TGFβ, the heightened activation of TGFβ signaling together with reduced TCF1, LEF1, BCL6 and ID3 favors aged T cell differentiation into TH9 cells [112]. 

Preferential development of a terminal effector cell state may explain the impaired vaccine response in old individuals [87]. After vaccination with the live varicella zoster virus (VZV) vaccine, generation of VZV-specific memory T cells is reduced in old adults [113]. Longitudinal analysis of VZV-specific CD4 T cells at effector and memory time points showed that reduced generation of memory T cells in old individuals resulted from accelerated attrition of effector T cells after the peak response [113]. Interestingly, transcriptome analysis on VZV-specific T cells at the peak of vaccine responses showed that reduced *TCF7* gene expression correlated with the greater contraction of VZV-specific T cells [109], supporting the notion that TCF1 is important for the generation of memory cells. Mechanistically, this bias toward terminal effector differentiation was in part due to the increased expression of miR-21 in old T cells [109]. miR-21 represses negative regulators of several signaling pathways including SPROUTY1, PTEN and PDCD4. As a result, proliferating old T cells had sustained activation of the ERK, AKT-mTORC1 and AP1 signaling pathways, which favored the differentiation of T cells to short-lived effector cells at the expense of memory precursors and TFH cells [109]. Antagonizing miR-21 reduced sustained activation signaling in differentiating old T cells, in turn upregulating expression of TCF1, LEF1 and BCL6 and inducing a memory precursor cell signature [109]. These data suggest that miR-21 or related pathways can be targeted to improve T cell responses in older individuals.

Favoring a gene regulatory network for effector differentiation can also be explained by increased expression of IL-2Rα in activated old T cells (Figure 3) [109]. Induction of BLIMP1 by IL-2/STAT5 signals represses TCF1 and BCL6 expression [23,29] and promotes generation of short-lived CD8 effector or terminal TH1 cells instead of memory precursors [31,39,40]. FOXO1 is one of downstream targets of the AKT kinase, and AKT-mediated phosphorylation of FOXO1 leads to its degradation [114]. FOXO1 is highly expressed in naïve T cells and downregulated after priming concomitant with the activation of AKT. During effector differentiation, FOXO1 expression was partially restored in young T cells, whereas differentiating old T cells had significantly less FOXO1, at least in part, due to sustained AKT activation [115]. FOXO1 is one of the transcription factors critical for the generation of memory CD8 T cells by repressing T-BET expression and promoting EOMES expression [45,46]. FOXO1 also binds to the *TCF7* promoter region and induces its expression [47,116]. Therefore, reduced FOXO1 expression by prolonged AKT activity is likely one of the mechanisms for reduced TCF1 expression in activated old T cells. Given that FOXO1 also regulates gene expression associated with cell survival [114], the precise contribution of FOXO1 to the differentiation of aged T cells remains to be determined. Interestingly, loss of FOXO1 activity is associated with the accumulation of short isoforms of TCF1 lacking the β-catenin binding domain [115]. Unlike functioning as presumptive dominant-negative regulators, TCF1 short isoforms were sufficient to support T cell maturation in the thymus [117] and memory CD8 T cell generation after acute infection [28], with long isoforms still needed for thymocyte survival and development of central memory phenotypes. Whether and how the altered expression of the different TCF1 isoforms influences aged T cell responses remains to be examined.

## 6. Conclusions

TCF1 controls common gene-regulatory networks governing the generation of TFH cells, memory precursor cells and stem-like CD8 T cells. By controlling transcriptional programs, TCF1 induces a stem-like cell phenotype, in part by repressing opposing cell fates including those of TH1 cells, short-lived terminal effector cells and terminally exhausted T cells (Figure 1). It remains to be determined whether TCF1 also influences mature T cell differentiation by controlling chromatin accessibility as it has been shown for thymic development [4]. At least in part, TCF1-dependent effects are due to the transcriptional expression of TCF1 as well as WNT signaling providing β-catenin as a transcriptional coactivator. It has been shown that WNT signaling contributes to the induction of a stem-like phenotype in CD8 T cell differentiation. Moreover, WNT signaling compensates for low TCF1 expression in the regulation of miR-181a expression that is reduced in naïve T cells from older individuals. The transcriptional control of TCF1 expression along T cell differentiation requires further studies. In early thymic development, NOTCH signaling is instrumental [4,118], however, whether the NOTCH pathway is also involved in controlling TCF1-dependent T cell differentiation as it does in thymic development is unclear. TCF1 expression declines with T cell activation, in part as a consequence of FOXO1 degradation.

The insights on the regulatory function of TCF1 in T cell differentiation are relevant for understanding the age-associated T cell dysfunction (Figure 2 and Figure 3) [119]. TCF1 is highly expressed in naïve T cells. With the age-related decline in TCF1 expression in naïve T cells, miR-181a expression decreases, leading to changes in T cell activation and differentiation. Reduced TCF1 expression with age may also be important for T cell homeostasis, in analog to being critical for memory T cell survival in acute infection as well as long-term persistence of exhausted CD8 T cells after chronic infection [52,120]. Moreover, dysregulated TCF1 expression is likely involved in the bias of old T cells to differentiate after activation into short-lived terminally differentiated effector cells over TFH or T memory cells. One mechanism for this bias is sustained AKT-mTORC1 signaling that results in prolonged repression of FOXO1 and consequently reduced TCF1 expression. This bias could explain the impaired T cell responses to infections and the ineffective vaccine efficacy seen in older individuals [88,121].

These insights provide new opportunities for the design of therapeutic interventions to improve T cell responses in older and even young individuals. Targeting TCF1 and TCF1-related regulatory networks could improve the generation and function of effector and memory T cells in responses to vaccination and infections in older individuals. Activation of WNT signaling could provide durable T cell function in controlling tumor growth [122]. Inhibition of AP1, AKT or mTORC1 could curtail sustained T cell activation and enhance the generation of memory T cells [123], likely by controlling the TCF1-BCL6/BLIMP1 axis.
